# In memoriam of Athineos Philippou

**DOI:** 10.1007/s00210-026-05432-4

**Published:** 2026-05-18

**Authors:** Helmut Greim, Roland Seifert

**Affiliations:** 1 Technical University of Munich, Munich, Germany; 2 Institute of Pharmacology, Hannover Medical School, Hannover, Germany

**Figure:**
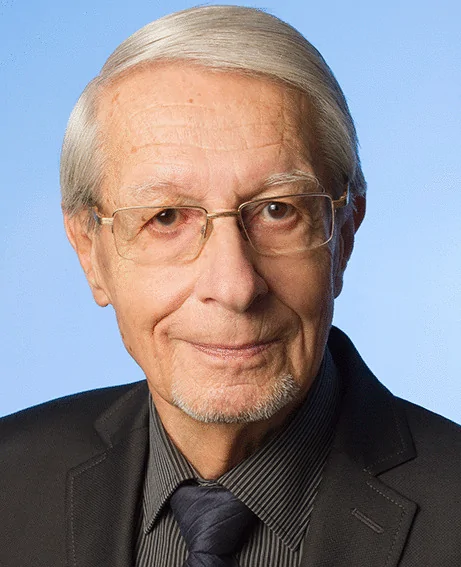

Athineos Philippou (Αθήναιος Φιλίππου) was born September 22, 1931, in Athens. His parents were the lawyer Ioannis Philippou and his wife Andromachi. Philippou attended schools in Athens and studied medicine there from 1950 to 1956. Alongside his medical studies, he worked on the cholinesterase activity of various leukocyte types and the chromatographic separation of carbohydrates as a student assistant and doctoral candidate at the Institute of Physiology at the University of Athens under Christos Maltesos, a student of Hermann Rein. In 1959, he received his doctorate in medicine by the dissertation “On the Cholinesterase Activity of Different Leukocytes”. He then served two and a half years in the Greek Navy. Thereafter, as a fellow of the Alexander von Humboldt Foundation during 1959 to 1962, he first worked at the Physiological Institute of the University of Cologne, and from 1960 on at the Institute of Pharmacology of the Johann Wolfgang Goethe University Frankfurt am Main, headed by Peter Holtz and subsequently became a research assistant. In 1965, he qualified as a university lecturer (Habilitation) in pharmacology and toxicology with a thesis entitled “Investigations into the Mechanism of the Storage and Release of Adrenal Medulla Hormones”.

When his Frankfurt mentor, the senior Holtz student Hans-Joachim Schümann, became director of the newly founded Institute of Pharmacology at the University Hospital Essen (now the University of Duisburg-Essen) in 1964, Philippou followed him.

In the group of Holtz and Schümann, Philippou turned his attention to the pharmacology of the nervous system. His research focused on storage granules (vesicles) that store the catecholamines norepinephrine and epinephrine in the cells of the adrenal medulla and in nerve cells. It was known that indirect sympathomimetics such as tyramine released the catecholamines. Schümann and Philippou investigated this further using isolated vesicles from the adrenal medulla. Tyramine released the catecholamines, but not the adenosine triphosphate stored with them, and at the same time, it was itself taken up into the vesicles. The group has shown that the release of catecholamines from chromaffin vesicles can be induced by tyramine, β-phenethylamine, amphetamine, methamphetamine, and ephedrine, but not by adenosine triphosphate. Furthermore, they showed that tyramine and β-phenethylamine accumulate in chromaffin vesicles in a stoichiometric ratio with catecholamine release and concluded that amphetamine and similar compounds act by displacing catecholamines without equivalent adenosine triphosphate release.

While still in Essen, Philippou first measured the release of noradrenaline in the hypothalamus of anaesthetised cats.

In 1968, he spent two months conducting research with Wilhelm Feldberg at the National Institute for Medical Research in London.

From 1970 to 1982, he was a professor and head of department under Ullrich Georg Trendelenburg at the Institute of Pharmacology and Toxicology at the Julius-Maximilians-University of Würzburg. With the move to Würzburg, research on the nervous system of living laboratory animals increasingly replaced research on cell organelles; neurochemical measurements continued to predominate using “push–pull cannulas” inserted into the brain consisting of two concentric tubes.

In 1982, he was appointed chair and founding director of the Institute of Pharmacodynamics and Toxicology, later renamed the Institute of Pharmacology and Toxicology, and since 1999, the Department of Pharmacology and Toxicology at the Institute of Pharmacy, Faculty of Chemistry and Pharmacy, University of Innsbruck, Austria. He retired in 1999 and continued to live with his family in Innsbruck, where he died January 28, 2026.

After his retirement as a pharmacologist in 1999, he started a second career as a historian. In 2001, the then chairman of the DGPT, Prof. K. Brune asked Philippou if he would be willing to document the history of pharmacology and toxicology. After some hesitation, he agreed and started to collected texts about institutes and images from pharmacologists and toxicologists. During more than 20 years, he collected the history of institutes and companies and gave prominent representatives of our field the opportunity to present their scientific work in the form of autobiographies. In addition, he persuaded authors to document the biographies of deceased colleagues. This comprehensive editorial work resulted in six volumes entitled “History and Activities of Pharmacological, Clinical-Pharmacological, and Toxicological Institutes in German-Speaking Countries”. These volumes provide a detailed history of these disciplines and a fundamental basis for their further scholarly exploration providing the younger generation the opportunity to learn something tangible for the field and for their own personal and professional futures.

In addition to these activities Philippou established the “Pharmakologie—Historisches Forum” within the annual meetings of the DGPT in 2014. The aim of the Forum is to honour personalities of Pharmacology, Clinical Pharmacology, and Toxicology of the various universities of Germany who, with their research, have greatly contributed to the development of their scientific disciplines. Further scope of the Forum is to inform young pharmacologists and toxicologists about lives and achievements of our predecessors and the history of Institutes located at the place of the actual annual meeting.

The first Forum was initiated by Roland Seifert and organized by Brigitte Lohff and Athineos Philippou and was held during the 80th Annual Meeting of the DGPT in Hannover. Specific topics, such as the Papyros Ebers, the ancient Egyptian guidance to diagnose and treat diseases from over 3000 years ago, have been presented in Leipzig (2020). These Forums have been very successful. After Philippou retired from this activity, the 10th Pharmacologic-Historical-Forum in Hannover (2025) has been organized by Roland Seifert, which covered various aspects of pharmacology during the NS regime from 1933 to 1945 including the fates and publications of persecuted pharmacologists, NS organization memberships of pharmacologist and the position of the German Pharmacological Society.

The 11th Forum in Düsseldorf 2026 has been organized by Helmut Greim and Roland Seifert. Wim Wätjen presented the academic life of Paul Ohnesorge, Nina Goebel *Lost voices: Tracing the unknown fate of Jewish members of the German Society of Pharmacology*, and Roland Seifert *Recently discovered photos tell a story: Meetings of German and British pharmacologists in England and Scotland between 1935—1939*.

The Universities of Helsinki and Innsbruck awarded Philippou their university medals in 1977 and 1999, resp. In 2008, he received the honorary doctorate from the Faculty of Medicine of the Aristotle University of Thessaloniki, Greece.

On March 10, 2022, the German Society for Pharmacology and Toxicology (DGPT) honoured Prof. Philippou with the Schmiedeberg Plaque for his outstanding contributions to document the highly eventful history of pharmacology and toxicology in German-speaking countries. The Schmiedeberg Plaque is the highest award bestowed by the DGPT awarded to scientists, who have made outstanding contributions to pharmacology and toxicology over decades.

Several of his most relevant publications are listed subsequently:

1. Philippu AJ (1956) Cholinesterase of leucocytes. Am J Physiol 184:145–146
2. Philippu AJ (1957) Cholinesterase content of the dog’s lymphocytes and polynuclears. Nature 180:438–438
3. Philippu A (1959) Separation of galactose, glucose and lactose in urine by paper chromatography. Anal Chem 31:1743–1744
4. Schümann HJ, Philippu A (1961) Untersuchungen zum Mechanismus der Freisetzung von Brenzcatechinaminen durch Tyramin. Naunyn-Schmiedebergs Arch exp Pathol Pharmakol 241:273–28
5. Schümann HJ, Philippu A (1962) Release of catechol amines from isolated medullary granules by sympathomimetic amines. Nature 193:890–891
6. Philippu A, Heyd G, Burger A (1970) Release of noradrenaline from the hypothalamus in vivo. Eur J Pharmacol 9:52–58
7. Philippu A, Przuntek H, Roensberg W (1973) Superfusion of the hypothalamus with gamma-aminobutyric acid. Effect on release of noradrenaline and blood pressure. Naunyn–Schmiedeberg's Arch Pharmacol 276:103–118
8. Philippu A, Matthaei M (1975) Uptake of serotonin, gamma-aminobutyric acid and histamine into synaptic vesicles of the pig caudate nucleus. Naunyn–Schmiedeberg's Arch Pharmacol 287:191–204
9. Robinson RL, Dietl H, Bald M, Kraus A, Philippu A (1983) Effects of short-lasting and long-lasting blood pressure changes on the release of endogenous catecholamines in the hypothalamus of the conscious, freely moving rabbit. Naunyn–Schmiedeberg’s Arch Pharmacol 322:203–209
10. Philippu A, Hagen R, Hanesch U, Waldmann U (1983) Changes in the arterial blood pressure increase the release of endogenous histamine in the hypothalamus of anaesthetized cats. Naunyn–Schmiedeberg’s Arch Pharmacol 323:162–167
11. Philippu A (1984) Use of push–pull cannulae to determine the release of endogenous neurotransmitters in distinct brain areas of anaesthetized and freely moving animals. In: C.A. Marsden (Hrsg.): Measurement of neurotransmitter release in vivo, S3–37. John Wiley & Sons Ltd
12. Klausmair A, Singewald N, Philippu A (1991) Release of endogenous catecholamines in two different regions of the nucleus of the solitary tract as influenced by carotid occlusion. Naunyn–Schmiedeberg’s Arch Pharmacol 343:155–160
13. Singewald N, Philippu A (1993) Catecholamine release in the locus coeruleus is modified by experimentally induced changes in haemodynamics, Naunyn–Schmiedeberg’s Arch Pharmacol 347:21–27
14. Schneider C, Singewald N, Philippu A (1995) Inhibition of catecholamine (noradrenaline, dopamine) release in the locus coeruleus and the hypothalamus by baroreceptor activation: identification of the involved baroreceptors. Naunyn–Schmiedeberg’s Arch Pharmacol 352:291–296
15. Philippu A (1988) Regulation of blood pressure by central neurotransmitters and neuropeptides. Rev Physiol Biochem Pharmacol 111:1–115
16. Prast H, Lamberti C, Fischer HP, Tran MH, Philippu A (1996) Nitric oxide influences the release of histamine and glutamate in the rat hypothalamus. Naunyn–Schmiedeberg’s Arch Pharmacol 354:731–735
17. Prast H, Grass K, Philippu A (1997 The ultradian EEG rhythm coincides temporally with the ultradian rhythm of histamine release in the posterior hypothalamus. Naunyn–Schmiedeberg’s Arch Pharmacol 356:526–528
18. Singewald N, Kouvelas D, Chen F, Philippu A (1997) The release of inhibitory amino acids in the hypothalamus is tonically modified by impulses from aortic baroreceptors as a consequence of blood pressure fluctuations. Naunyn–Schmiedeberg’s Arch Pharmacol 356:348–355
19. Prast H, Tran MH, Lamberti C, Fischer HP, Kraus M, Grass K, Philippu A (1999) Histaminergic neurons modulate acetylcholine release in the ventral striatum: role of H_1_ and H_2_ histamine receptors. Naunyn–Schmiedeberg’s Arch Pharmacol 360:552–557
20. Philippu A and Prast H (2001) Role of histaminergic and cholinergic transmission in cognitive processes. Drug News & Perspect 14:523–529

